# The Destruction of Rats

**Published:** 1901-10-12

**Authors:** 


### EDITOR'S LETTER-BOX.

[Our Correspondents are reminded that prolixity is a great bar to publication, and that brevity of style and conciseness of statement greatly facilitate early insertion.]

### THE DESTRUCTION OF RATS.

*To the Editor of* The Hospital.

Mr. P. Vernon Kays, of Kay Brothers, Stockport, writes:—

In reference to the article which appeared in your last week's issue on "Plague and its Prevention" we notice that medical opinion is practically unanimous in holding that the common rat plays an important part in the spreading of infection.

It is curious that in the many letters that have appeared in the press on this subject little or no reference has been made to what is one of the simplest methods of catching these pests. We refer to the use of birdlime. In almost all parts of the world, especially in Japan and the Far East, this article is in common use for the purpose; the fact of it being non-poisonous is a great point in its recommendation. We are not writing this letter for the purpose of advertisement, although we have for many years dealt in this article, but in the hope that this letter may be of service to those who are investigating the cause of the spreading of the disease. Rats and mice are thus caught and held for examination, and do not, as in the case when poisons are used, crawl off to their holes and die in inaccessible places. If we can give you any further information on this point we shall be pleased to do so. We have been interested in this line for some 35 years.

